# “I would love to see these big institutions… throwing their weight around”: qualitative findings regarding health and social sector collaborations to address community-level socioeconomic adversity

**DOI:** 10.1186/s12889-024-19465-y

**Published:** 2024-07-29

**Authors:** Erika M. Brown, Taressa K. Fraze, Laura M. Gottlieb, Caroline Fichtenberg

**Affiliations:** 1 California Policy Lab, Institute of Research on Labor and Employment, University of California, Berkeley, 2521 Channing Way, Berkeley, CA 94704 USA; 2 Department of Family and Community Medicine, University of California, San Francisco, 995 Potrero Ave, San Francisco, CA 94110 USA

**Keywords:** Socioeconomic adversity, Social determinants of health, Social care, Cross-sector partnerships, Social sector, Advocacy

## Abstract

**Background:**

Health and social sector organizations are increasingly working together to mitigate socioeconomic adversity within their communities. We sought to learn about the motivations, experiences, and perspectives of organizations engaged in these collaborations.

**Methods:**

We conducted semi-structured, 60-minute interviews with 34 leaders from 25 health and social sector organizations between January-April 2021. Interviews explored motivations, benefits and challenges, and ways in which health sector organizations can most effectively address community-level socioeconomic adversity. Interviews were audio recorded and transcribed; themes were coded using Dedoose software.

**Results:**

Partnerships were primarily motivated by mission-driven organizations and key health sector leaders who were interested in addressing root causes of poor health; policies such as certificate of need laws and value-based care incentives that aligned community-level investments with health sector organizations’ financial interests facilitated these efforts. While partnerships were mostly regarded as mutually beneficial ways to increase impact (for the health sector) and resource access (for the social sector), social sector organizations voiced frustrations regarding the outsized expectations, unsustained interest, and lack of partnership from their health sector collaborators. Despite these frustrations, both health and social sector interviewees supported the health sector’s continued involvement in community-level socioeconomic initiatives and expansion of policy and systems efforts.

**Conclusions:**

Cross-sector, community-level socioeconomic initiatives were mutually beneficial, but social sector organizations experienced more frustrations. Policy and organizational changes within the health sector can further mobilize and sustain support for these efforts.

**Supplementary Information:**

The online version contains supplementary material available at 10.1186/s12889-024-19465-y.

## Background

United States health care policies are increasingly incentivizing – and in some cases, requiring – payors and providers to integrate activities that identify and aim to address social adversity into clinical care [1]. Many health sector organizations have responded by implementing social risk screening and referral programs for their patient and member populations [2–6]. A number have chosen to focus their attention further upstream by tackling the community conditions that give rise to social needs [7–10]. For example, some health sector organizations are providing financing for affordable housing and economic development projects; [11–14] hiring and procuring locally to strengthen the local economy; [15] and lending political capital and operational support to socioeconomic policy and systems change efforts [16, 17]. These efforts could be beneficial to communities, adding resources and sway to issues that often lack both. However, such initiatives also use financial resources that could be allocated elsewhere and often require new skills and relationships. It is not yet clear whether these activities will remain confined to a select group of mission-oriented organizations, or whether others might also be motivated or incentivized to put their resources to use in these ways.

Further, social sector organizations are often critical to the long-term success of the health sector’s community-level efforts. Their extensive experience could help health sector organizations more strategically invest and deploy resources [18]. Several studies have explored how social sector organizations perceive the health sector’s interest in addressing socioeconomic adversity, [19–22] but the authors have primarily focused on referral-based initiatives that strictly target healthcare patient and member populations. To our knowledge, no studies have explored whether social sector organizations welcome the community resources that the health sector brings or resent the intrusion of a sector with limited experience in issues such as housing, food assistance, and economic development. More evidence is needed to understand social sector leaders’ perspectives on if and how they envision impactful roles for health sector organizations hoping to reduce socioeconomic challenges in their communities.

To help answer these questions, we interviewed leaders from health and social sector organizations that were jointly implementing efforts to address socioeconomic adversity at the community-level to understand the motivations, experiences, and perspectives from both sides of these collaborations. This paper aims to inform health sector organizations seeking to engage in community-level social determinants of health work – particularly those involving collaborations with social service sector organizations – as well as policy makers interested in incentivizing effective health sector community investments and actions.

## Methods

### Study design

From January to April 2021, we interviewed health and social sector organization leaders as part of the *Raising the Bar* project, a Robert Wood Johnson Foundation-funded effort led by the National Alliance to impact the Social Determinants of Health. The goal of *Raising the Bar* was to develop principles and provide practical guidance for the health sector to achieve optimal well-being for individuals facing the greatest barriers to health [23]. Study activities received an exemption determination from the University of California, San Francisco Institutional Review Board.

### Recruitment

We leveraged prior knowledge, field experts, and internet searches to identify health sector organizations (including payors, providers, and coalitions) and social sector organizations (including direct service providers, advocacy organizations, and coalitions, among others) that were jointly engaged in cross-sector, community-level activities to address socioeconomic adversity. Joint engagement was defined as any level of collaboration, ranging from one-time funding efforts to multi-year partnerships. Community-level activities were defined as initiatives that sought to benefit individuals in a given region, regardless of whether they were part of a health sector organization’s patient or member population. Activities had to focus on addressing socioeconomic issues (e.g., a lack of affordable housing or high unemployment). We identified 14 collaborations that were diverse in regard to their targeted social condition(s), programming, and geography, as we aimed to represent multiple regions of the United States.

We e-mailed the contact or intervention lead (as determined by websites, reports, manuscripts, personal contacts, and collaborating organization(s)) to request an interview with them, or the individual(s) they thought could best speak to their cross-sector activities and collaboration(s). Of the 31 organizations we contacted, individuals from 25 agreed to participate in our study, representing a total of 11 collaborations. We spoke with at least one health sector organization and one social sector organization contact from nine initiatives; we were only able to speak with one health or social sector organization from two initiatives because we were unable to successfully contact their collaborator(s). All organizations were interviewed separately to ensure that interviewees could speak privately and freely about their experiences.

### Data collection

Two study team members (EMB, CF) conducted semi-structured interviews over video calls that typically lasted 60 min. One to three individuals from each organization participated in each call; all participants were offered a $50 honorarium for their time. The interviews covered descriptions of the community-level socioeconomic efforts, reported or perceived motivations for the health sector to engage in community-level efforts, facilitators and barriers to successful cross-sector collaborations, as well as the roles that both types of organizations would like to see the health sector play in addressing socioeconomic adversity at the community level (see Supplementary material for the interview guide). Interviews were recorded and professionally transcribed.

### Analysis

We used an iterative, inductive analytic approach [24] to establish themes within and across sectors. Two study team members (EMB, CF) read all transcripts, surfaced patterns, and discussed developing themes during weekly meetings. These themes were used to create an initial codebook, which was applied to three transcripts by both team members. A third team member (TKF) helped to refine the final codebook, which was then blindly applied to three more transcripts by the same coding team (EMB, CF). After consensus regarding the content and application of each code was reached, one team member (EMB) applied codes to the remaining transcripts. All coding was conducted using Dedoose software.

We summarized codes in several spreadsheets using a framework matrix [25]. This orientation allowed us to determine the frequency of each theme and identify patterns across organizations and by organizational type (health vs. social sector). We then developed a detailed memo of the results with examples to support each theme. Three study team members (EB, CF, TKF) met weekly to discuss the analysis until consensus regarding each theme was reached.

## Results

### Characteristics of participant and community-level socioeconomic initiatives

Our final sample included 34 individuals from 25 organizations (12 health sector organizations and 13 social sector organizations) involved in 11 collaborations. Most organizations served specific regions of the United States (predominantly the Midwest) and four operated nationally. Seven of the 12 health sector organizations were health systems or hospitals, three were payors, and two were health coalitions. The social sector organizations included 4 direct service providers and 3 policy or systems change advocacy groups. More details about the organizations and collaborations can be found in Table 1.

**Table 1:** Characteristics of a sample of health and social organizations engaged in cross-sector, community-level, socioeconomic initiatives

Region	Project(s)	Org	Organization type	Interviewee Title(s)
Western state	Evidence building as a form of advocacy: tailored food distribution for families, facilitating community connections to housing	1	HSO: Health care delivery system	Manager/Program Operations
				Executive
		2	SSO: Advocacy organization	Director
		3	SSO: National non-profit organization	Director
Northwestern metropolitan area	Grant-funded investments in affordable housing; coalition building	4	HSO: Health care payor	Manager/Program Operations
				Manager/Program Operations
		5	HSO: Integrated health care delivery system	Director
				Manager/Program Operations
		6	SSO: Community-based service provider	Executive
Midwestern metropolitan area	Local hiring and procurement, multi-sector coalition building	7	HSO: Academic affiliated, non-profit health care delivery system and workforce coalition	Manager/Program Operations
				Manager/Program Operations
				Manager/Program Operations*
		8	SSO: Workforce development organization	Manager/Program Operations
Midwestern metropolitan area	Evidence building as a form of advocacy, building and renting affordable units; home repair	9	HSO: Academic affiliated, non-profit hospital	Director
				Director
		10	SSO: Community-based direct service provider	Executive
		11	SSO: Local philanthropic organization	Manager/Program Operations
Midwestern metropolitan area	Local hiring and procurement	12	HSO: Health system	Director
		13	SSO: Workforce development organization	Executive
Midwestern metropolitan area	Transportation advocacy	14	HSO: Multisector community health coalition	Director
Southern metropolitan area	Creating a retail space for healthy, affordable prepared meals and produce	15	HSO: Public, academic affiliated, safety net health system	Manager/Program Operations
		16	SSO: Community-based service provider	Executive
Southern metropolitan area	Multi-sector coalition building; integration of health and social service organizations	17	HSO: Multisector health coalition	Director
				Director
				Director
Southeastern state	Evidence building as a form of advocacy; community SNAP enrollment	18	HSO: Health care payor	Executive
		19	SSO: Community-based organization	Executive
Northeastern metropolitan area	Funding and facilitating community connections to housing	20	HSO: Academic affiliated safety net health system	Director
				Manager/Program Operations
		21	SSO: Community-based service provider	Manager/Program Operations
Nationwide	Advocacy for affordable housing: legislative efforts and awareness raising	22	HSO: Healthcare coalition	Director
		23	SSO: Multisector advocacy campaign	Director
		24	SSO: Advocacy organization	Director
		25	HSO: Health system	Director

*Played a substantial role in community-level coalition

HSO = Health sector organization; SSO = Social sector organization

### Factors motivating health sector organizations to engage in community-level socioeconomic initiatives

Health sector organizations were primarily motivated by mission and/or self-interest. The mission-driven organizations spoke to the importance of improving health and wellbeing within their communities, emphasizing the limitations of siloed clinical care. As one physician leader shared: “*I was doing research mostly on why children in poverty had such poor outcomes no matter what condition we looked at. And got very frustrated that some of my colleagues had been seeing the same families*,* generation after generation of clinics*,* with no real change. And I knew there had to be a better way*.” These organizations believed that more sustained, community-level collaborations were critical tools for addressing the structural and socioeconomic factors that gave rise to poor outcomes within their communities. One health sector executive relayed, “*to [achieve] health equity… we can’t do it solely by ourselves. We need to do it through innovative partnerships in the community. We need to look beyond traditional medical models and address social determinants of health*.”

Interviewees also noted a myriad of direct benefits that extended beyond improved capacity to achieve their mission. One shared that improving the safety and quality of life in their local community would better enable them to recruit top talent; another believed it improved employee morale. Others expressed that they wanted to improve their reputation within and beyond the populations they served. One interviewee said: *“So*,* I’m not saying [good public relations] is why people do things… [our leadership] like[s] this stuff*,* and they love doing things that help people*,* but they’re doing these things for a reason. And the reason is it’s good for business.”* Two interviewees noted that the direct connection between broader socioeconomic conditions and their bottom line motivated them to act. As one health payor executive explained: “*Not only is [socioeconomic adversity] costly for people who are our members*,* but people who will be our members next year will come into our organization with higher potential medical costs if we don’t address [it] within their communities.*” Many other interviewees shared that value-based care and other financial incentives were key facilitators – if not primary motivators – for executing projects.

Some collaborations or specific projects were catalyzed by local requirements (e.g., needing to meet a formal or informal community benefit requirement related to capital projects) or findings from Community Health Needs Assessments. Feedback from local partners also helped motivate actions on socioeconomic adversity by raising health sector organizations’ awareness of the health-related socioeconomic needs of their communities, as well as specific issues communities wanted to prioritize.

Critically, almost all the interviewees we spoke with noted that at least one mission-driven leader at their organization was responsible for either introducing their initiative(s) or moving them forward. Most were executives who set institution-wide priorities from the top, but several were high profile researchers who leveraged a requirement, policy, or structural change within their region or the broader health sector (e.g., their organization’s transition to value-based care) to push for their community-level collaboration.

### Benefits of health–social sector collaborations

Many health sector organization interviewees spoke directly to how partnering with social sector organizations enabled them to expand their programmatic reach and engage with their communities more effectively than they could on their own. Specifically, they appreciated social sector organizations’ content expertise, pre-existing relationships and trust built within their communities, as well as how working together allowed them to reduce duplication of services across different sectors.

For interviewees from social sector organizations, increased resource access was the motivator for engaging with health sector organizations, particularly the potential for health sector organizations to become long-term funders of their work. Those in the social sector also highlighted additional capacities and non-financial resources that health sector organizations could provide, such as public relations or research capacity, the latter of which they hoped could be used to make the business case for sustaining their joint initiatives or catalyzing policy changes.

Additionally, several social sector organizations expressed that connecting their work to health-centered goals and language (e.g., using the term “social determinants of health”) helped increase the perceived legitimacy of their work. One director said: “*I think there’s a lot of respect for health care across the political spectrum. So when a group like the American Academy of Pediatrics says we need housing vouchers*,* people are going to pay attention to that.*” An executive also shared: “*We found that [working with our healthcare partner] gave us knowledge*,* expertise*,* and vocabulary that helped us to articulate what we were up to in a way that allowed us to move beyond the circles where we started.”*

### Challenges of health–social sector collaborations

Social sector organizations voiced several key challenges and frustrations about their collaborative efforts with the health sector. Interviewees shared that health sector organizations’ funding and interest were unreliable, that they did not invest as much into understanding social sector organizations or the scope of the issues they were trying to address, were not transparent about their decision-making processes, and did not compromise or meaningfully listen to their collaborator’s priorities. One direct service provider said, *“I’ve seen very few healthcare partners that are willing to truly meet us in the middle*,* rather than just think that this is all a good idea*,* but we need to do all the work to make it easy for them*.”

Two social sector organizations noted that health sector partners underestimated the resources required to meaningfully impact socioeconomic adversity, leading to outsized expectations that they could not meet. One interviewee shared: “*I think [our healthcare grant] came from a place of wanting to fund and build on infrastructure… but it still ended up being a drop in the bucket. The hospitals want to know why they can’t refer their patients*,* and I’m like… if you want to refer every person who’s rent-burdened in the city for resources*,* you’re talking about half the people who live here.”*

Since health sector organizations’ efforts were often pushed forward by just one or two key leaders, interviewees from social sector organizations expressed concerns that their collaborations were vulnerable to leadership changes. Some also highlighted the fact that senior leadership support for these non-clinical initiatives did not always translate into sustainability, especially when the initiatives were not reflected in institutional goals and metrics. One executive shared, *“it’s great that you have the people on top saying*,* ‘we want to do this*,*’ but the reality in big organizations is that the day-to-day people are making the decisions*,* not the CEO. What the CEO says doesn’t necessarily always agree… with what their objectives are… and what their metrics are.”*

Notably, most of the aforementioned challenges raised by social sector organizations were expressed by interviewees engaged in grant- or contractor-based relationships. Social sector organizations that had short-term, low commitment asks (e.g., national advocacy organizations seeking a statement for a specific campaign) or shared governance of their initiatives reported fewer issues. Expectations of health sector collaborators in these arrangements were either uniquely limited or more consistently met; health sector organizations that institutionalized their initiatives or formalized equal partnerships dedicated more resources, an assurance of sustainability, and effort towards making both their relationship with the social sector and their intervention(s) work.

Health sector organizations in grant- or contractor-based relationships were also most vocal about challenges within their collaborations. A few interviewees working with direct service providers felt constrained by smaller social sector collaborating organizations that couldn’t scale across their organization’s footprint. Some shared that organizational resources and internal support for their work were lacking, while at the same time they were faced with pressure to demonstrate short-term returns on investments despite limited capacity for evaluation and the fact that community-level initiatives often have long-term horizons. Even interviewees from organizations that had committed to institutionalizing their community-level initiatives lamented how their priorities either didn’t reach all departments or weren’t universally shared. Many expressed that their collaborations competed for the time, resources, and attention that they needed to allocate towards providing or paying for health care, creating a tension between their official charge as providers or payors and community-level collaborative efforts. One leader expressed: “*I think health systems feel like we get asked to do everything*,* and we can’t do everything… [we’re] also trying to run health systems.”*

### Recommended roles for health sector organizations in community-level activities

Despite these challenges, interviewees from both types of organizations unanimously believed that health sector organizations should contribute to addressing community-level socioeconomic adversity given (1) the substantial links between socioeconomic adversity and health, and (2) the health sector’s financial resources and influence relative to the social services sector. Social sector organizations highlighted what they saw as particularly impactful roles for health sector organizations, including providing funding, supporting research to build the evidence for select activities, and providing in-kind assistance with activities to support community-level projects (e.g., public relations and grant writing). Additional recommendations regarding the roles and responsibilities for health sector organizations are described in Table 2.

**Table 2:** Recommended activities for health sector organizations interested in intervening upon community-level socioeconomic adversity

**1. Leverage financial assets**
• Serve as large-scale investors, e.g. provide loans to promote affordable housing • Provide sustainable funding streams to direct social service organizations for contracted services and socioeconomic initiatives (e.g., food as medicine)
• Provide large, lump-sum grants for project development
• Pay for study implementation and evaluation to build evidence/proof of concept
• Create staff positions to facilitate social determinants work
2. **Provide in-kind support to community-improvement efforts**
• Provide in-kind support for grant-writing, real estate transactions, marketing, communications, etc.
3. **Change internal policies and culture to facilitate local hiring and procurement efforts**
• Reconsider exclusion of individuals with history of incarceration
• Eliminate irrelevant education barriers (e.g., high school equivalent for entry-level janitorial positions)
• Provide on-site supports for new hires and create welcoming environment that reflects understanding of diverse lived experiences
• Redefine professionalism from a non-white, non-patriarchal lens
• Provide a living wage to employees
• Create racial equity hiring targets
4. **Advocate for social policy and systems changes**
• Meet with local district and congressional members to push for issues related to social determinants
• Advocate for specific pieces of legislation
• Use external communications and influence to raise issue awareness
5. **Participate in and support coalition building**
• Thoughtfully and meaningfully partner with social sector organizations
• Build public/private coalitions
• Engage with the community

Although social sector interviewees appreciated the financial resources that the health sector contributed to their work, many specifically mentioned wanting healthcare organizations to more actively advocate for systems and policy change, which they believed would most effectively influence key issues such as housing affordability. One social sector organization shared: *“The housing crisis requires a large-scale policy solution … there are all sorts of laudable efforts that we’ve seen over the past many years from health groups that have been investing a few million dollars in new affordable units. That’s fantastic*,* but we are not going to solve the nation’s housing crisis program by program*.” Another expressed, “*I would love to see these big institutions play more of a role*,* throwing their weight around to generate housing.”* In addition to being a more impactful approach in the long term, health sector advocacy for non-health issues could also provide them with a direct benefit, as one former health sector leader highlighted: “*[By advocating for housing] I think that not only were we … moving an issue forward that is fundamentally related to a person’s health*,* but at the same time*,* I thought we were building equity with members of Congress by really demonstrating*,* ‘Look*,* we care about more than just our bottom line.’ I thought it was strategically just really great for future relationships.”*

Several social sector organizations and health sector organizations also explicitly noted that health sector initiatives should be created and implemented in conjunction with community partners: “*[Healthcare organizations’] role would be co-creating… it’s almost like a new approach to the way we do health*,* beyond healthcare. And there’s so much that’s happening on a national stage in there*,* and there’s so much opportunity to develop an effective system that actually meets the needs of community members. To make it work*,* they’ve got to be a partner in that. Otherwise*,* it will just become another silo.*” Social sector interviewees cautioned that health sector organizations interested in pursuing community-level work with cross-sector partners needed to enter relationships with humility and a desire for shared decision making. To that point, one coalition leader shared: *“I would say [the ideal role for health sector organizations] really needs to be focused on the listening.”*

## Discussion

We spoke with a sample of health and social sector organizations to better understand their experiences and perspectives regarding cross-sector community-level socioeconomic initiatives. Interviewees unanimously agreed that health sector organizations should leverage their financial and non-financial capital to bolster work being done by the relatively resource-poor social sector. One unexpected finding relates to the number of interviewees who mentioned wanting health sector organizations to increase their policy and systems change efforts. This seemed to be motivated by two beliefs: (1) that only policy and systems changes will be large enough to address the socioeconomic challenges facing communities; and (2) that health sector organizations possess substantial political power that could influence policy debates. The perceived lack of focus on policy change among health sector organizations is consistent with the results of a 2021 study that found that the 10 health care organizations that spent the most on federal lobbying from 2015 to 2019 did relatively little lobbying related to social determinants, and none related to housing issues [16]. Taken together, these findings highlight that policy and systems change efforts may be an area where more health sector organizations – even those with limited financial resources or partnership forging capacity – could substantially increase their activities.

However, interviewees underscored numerous challenges that arose from joining organizations with disparate core functions and incentives. These results are congruent with prior findings suggesting that social service organizations, although enthusiastic about the potential benefits of partnering with health sector organizations, are wary of the power imbalance and mission misalignment that exist between the two sectors. [19–21] This concern has been voiced by some within the health sector as well. [26] Several actions may shift the power imbalance for organizations interested in collaborative community-level work. Increasing shared governance through bi-directional board membership may help to ensure that community voices are central to the development and implementation of joint initiatives, and that health sector leaders are keyed into critical issues facing their partners. Power sharing can also be baked into contracts and agreements. At an interpersonal level, health sector organizations can better respect the expertise of social service professionals, listen more carefully to their goals and priorities, and invest more time and energy into understanding their cultures and needs.

At a policy level, the health sector can better align their work with that of the health sector by strengthening existing policies and regulations to increase the adoption and sustainability of community-driven socioeconomic initiatives. We found that this work was largely driven by mission-driven health sector leaders and organizations, but policies and structural facilitators – such as community benefit requirements, community health needs assessments, and value-based care incentives that aligned health sector organizations’ financial bottom line with their community’s wellbeing – were key catalysts. Stronger community-focused or more deliberately structured requirements could enable more health organizations to effectively marshal resources without relying on the political will of select leaders or overextending the sector. For example, state policymakers can provide metrics to direct spending on specific activities could strengthen the role of not-for-profit hospitals in promoting community-level socioeconomic wellbeing [27]. At least four states (Arizona, Nevada, Oregon, and Tennessee) already require Medicaid managed care organizations to invest a percentage of their annual profits into community organizations and/or activities. [28] Certificate of Need laws – regulatory mechanisms that exist in 35 states and the District of Columbia for approving major capital expenditures and projects for some health care facilities [29] – can also promote community investment more deliberately among hospitals and healthcare systems aiming to expand their physical footprints.

These findings should be interpreted in light of their limitations. We recruited from a purposive sample of organizations with known initiatives, and selected sites based on the existence of a health/social sector collaboration. These collaborations are inherently more likely to reflect the experiences of organizations that found ways to make their relationships and endeavors work. While we missed learnings from organizations that attempted and failed at developing collaborations, which could have offered valuable lessons, focusing on successful collaborations allowed us to identify effective strategies for engaging in this work. Moreover, they still provided insights into less successful strategies and challenges. In addition, our sample was not representative: we interviewed only 1–3 individuals from 25 organizations that were disproportionately concentrated in the Midwestern region of the United States, limiting the generalizability of our results. Nevertheless, these findings are some of the first to explore, and to our knowledge, among the first that highlight perspectives of social sector organizations about, health sector organizations’ community-level socioeconomic initiatives.

Future studies can build on this work by exploring how organizational structures, environments, and motivations influence the trajectory of community-level interventions, incorporating a broader range of collaborations that failed or did not come to fruition. Applying organizational theory to data collection and analysis would provide more detailed insights regarding the adoption, implementation, and success of different interventions across the health and social sectors, including their impacts on population health and health equity.

## Conclusion

Our study is among the first to explore health and social sector organizations’ perspectives on their collaborative activities aimed at addressing socioeconomic adversity at the community-level. We found that these efforts are facilitated by policies that make these kinds of investments align with health sector organizations’ business interests and that highlight communities’ socioeconomic needs. Despite challenges resulting from the power imbalance and cultural differences between health and social sector organizations, both health and social sector interviewees supported the health sector’s continued involvement in community-level socioeconomic initiatives. Interviewees’ particular emphasis on policy and systems changes suggest the health sector is currently underinvested in that strategy to improve community health.

### Electronic supplementary material

Below is the link to the electronic supplementary material.

Supplementary Material 1

## Data Availability

No datasets were generated or analysed during the current study.
