# Supplementary material for: “I would love to see these big institutions… throwing their weight around”: qualitative findings regarding health and social sector collaborations to address community-level socioeconomic adversity
Source: BMC Public Health. 2024 Jul 29;24:2020. doi: 10.1186/s12889-024-19465-y (PMC11285253; doi:10.1186/s12889-024-19465-y)
Supplement: Supplementary file 1 — Supplementary Material 1 [file 12889_2024_19465_MOESM1_ESM.docx]

| **Background (2-4 minutes)**: Can you explain your role at [organization]? How long have you worked there? |
| --- |
| **Primary domains (50 minutes):** |
| *How is your organization working with [ collaborating organization] to meet the social needs of your population at the community-level?*  We are hoping to learn how inter-sector partnerships and efforts are successfully promoting health equity and addressing social needs within your community, both in the past and present. We are particularly interested in understanding where and how you believe healthcare organizations can make the greatest impact.  To start, can you give me an overview of any current partnerships and collaborative efforts between your organization and *[collaborating organization]*?  **Activity probes (20 minutes)**   - - **What is the goal of [described activity]? What needs does it target? What strategies does it employ?**   - **What has your role been in [described activity]?**   - **What is the role of [partnering organization] in [described activity]?**   **How did [described activity] arise?**  Who in the organization champions it?  What does senior leadership think about it?   - - - *For health sector organizations only*: What motivated your organization to engage with this work?   - **How has the [activity] gone? What’s worked? What hasn’t?**   - Did external pressures influence this work? How?     - Internal pressures?   - How has this work been funded?   - How has COVID changed the nature of these activities and your organization’s priorities?   **Collaboration probes (15 minutes)**   - - How and why did the relationship with [**partnering organization]** develop?     - What incentivized your collaboration?     - More collaborators in the pipeline?   - **How has [partnering organization] helped to facilitate the goals of [described activity]? How did partnering make it more difficult?**   - What is your goal/vision for the collaboration? How was it established?   - What has and hasn’t worked well?   What has surprised you about the collaboration?   - - What would you do differently?   **Health sector organizations probes: to be asked of all parties (15) minutes)**   - - **How can health sector organizations provide the most value to community-level social interventions?**     - **What is [their] ideal role in the context of existing work?**     - **What is [their] Ideal role in the absence of existing work?**     - **Should they tackle specific social needs? All needs?**   **How can health sector organizations best prevent duplication of existing work? Or**  **How can health sector organizations best prevent unintended consequences?** |
|  |
|  |
| **Future work (5 minutes):**  Are there any additional activities in the pipeline?  What advice do you have for others embarking on similar work or partnerships? |
